# Clinical Research on Type 2 Diabetes: A Promising and Multifaceted Landscape

**DOI:** 10.3390/jcm11206007

**Published:** 2022-10-12

**Authors:** Fernando Gómez-Peralta, Cristina Abreu

**Affiliations:** Unidad de Endocrinología y Nutrición, Hospital General de Segovia, 40002 Segovia, Spain

Type 2 diabetes constitutes an imposing epidemiological, economic, and scientific global challenge. The chronic complications of type 2 diabetes are a major cause of mortality and disability worldwide [1,2]. Clinical research is the main way to gain knowledge about long-term diabetic complications and reduce the burden of diabetes. This allows for designing effective programs for screening and follow-up and fine-targeted therapeutic interventions. However, new research methodologies are needed to obtain more accurate and useful insights into the biological and clinical processes involved in diabetic complication development.

During the last few years, new approaches for clinical research have incorporated digital tools to analyze the complex physiopathological background of type 2 diabetes. In this *Special Issue,* entitled “*Clinical Research on Type 2 Diabetes and Its Complications*” and published in the *Journal of Clinical Medicine* (https://www.mdpi.com/journal/jcm/special_issues/Type_2_Diabetes_Complications), some valuable digital methodologies were used in different studies focusing on the type 2 diabetes syndrome. Novel machine learning techniques for predicting long-term complications are one of these approaches, as the studies of Huang, Rashid, and Shin et al. depict [3,4,5]. The data presented by these authors suggest that machine learning may be more accurate in predicting diabetic microvascular complications than traditional methods. Additionally, digital tools such as artificial intelligence and machine learning can be implemented through an automated and rapid process.

Among the frequent causes of frustration for people with diabetes and the health care providers involved in their management is the delayed detection of diabetic complications. The outlook of clinical research appears promising in the near future owing to the development and implementation of advanced methods for the detection of early alterations in the micro- and macrovascular complications associated with diabetes. Two papers in this *Special Issue* cover the use of specific biomarkers tracing the progress of diabetic cardiovascular complications [6,7]. In another contribution, Lee et al. revisit the long-term glycemic variability and its relationship with end-stage kidney disease [8].

Besides the genetic approach, the application of digital techniques, including machine learning and artificial intelligence, and novel biomarkers could be crucial for individualized type 2 diabetes management, which is the backbone of precision medicine.

Two review papers address the complications that are non-traditionally linked to type 2 diabetes, although currently under exhaustive research: bone health and non-alcoholic fatty liver disease [9,10]. The multifaceted nature of type 2 diabetes is clearly visualized owing to the holistic angle used by these approaches.

The efficacy and safety of new type 2 diabetes pharmacological treatment are covered by three original papers [11,12,13]. The Yu-Chuan Kang et al. study includes a large population sample and an extended follow-up to evaluate the association between dipeptidyl peptidase-4 inhibitors and diabetic retinopathy [13]. This could be the first signal for a new safety risk of a pharmacological class of drugs used by millions worldwide.

The COVID-19 pandemic was first reported in China in December 2019 and continues to be a devastating condition for global health and economy. The COVID-19 disease has immediate implications for common chronic metabolic disorders such as type 2 diabetes. Both direct infection and the associated distress due to preventive measures in the general population have worsened the control of type 2 diabetes. Some factors indicate that COVID-19 or other coronavirus-caused diseases can be seasonal or persistent in the future. Type 2 diabetes has a strong negative effect on the prognosis of patients with COVID-19. Three papers in this *Special Issue* review the implications of this disease in relation to diabetes [14,15,16].

Finally, the aim of researchers in this field should be to make all these remarkable advances accessible to those populations experiencing more difficulties due to sociodemographic factors such as cultural deprivation, sex discrimination, or limited income [17,18,19].
